# High frequency accuracy and loss data of random neural networks trained on image datasets

**DOI:** 10.1016/j.dib.2021.107780

**Published:** 2022-01-05

**Authors:** Ariel Keller Rorabaugh, Silvina Caíno-Lores, Travis Johnston, Michela Taufer

**Affiliations:** a University of Tennessee, Knoxville, TN 37996, USA; b Striveworks, Austin, TX, USA

**Keywords:** Loss curve, Accuracy curve, Classification, Performance prediction, Early stopping, Neural architecture search, Machine learning, Artificial intelligence

## Abstract

Neural Networks (NNs) are increasingly used across scientific domains to extract knowledge from experimental or computational data. An NN is composed of natural or artificial neurons that serve as simple processing units and are interconnected into a model architecture; it acquires knowledge from the environment through a learning process and stores this knowledge in its connections. The learning process is conducted by training. During NN training, the learning process can be tracked by periodically validating the NN and calculating its fitness. The resulting sequence of fitness values (i.e., validation accuracy or validation loss) is called the NN learning curve. The development of tools for NN design requires knowledge of diverse NNs and their complete learning curves.

Generally, only final fully-trained fitness values for highly accurate NNs are made available to the community, hampering efforts to develop tools for NN design and leaving unaddressed aspects such as explaining the generation of an NN and reproducing its learning process. Our dataset fills this gap by fully recording the structure, metadata, and complete learning curves for a wide variety of random NNs throughout their training. Our dataset captures the lifespan of 6000 NNs throughout generation, training, and validation stages. It consists of a suite of 6000 tables, each table representing the lifespan of one NN. We generate each NN with randomized parameter values and train it for 40 epochs on one of three diverse image datasets (i.e., CIFAR-100, FashionMNIST, SVHN). We calculate and record each NN’s fitness with high frequency—every half epoch—to capture the evolution of the training and validation process. As a result, for each NN, we record the generated parameter values describing the structure of that NN, the image dataset on which the NN trained, and all loss and accuracy values for the NN every half epoch.

We put our dataset to the service of researchers studying NN performance and its evolution throughout training and validation. Statistical methods can be applied to our dataset to analyze the shape of learning curves in diverse NNs, and the relationship between an NN’s structure and its fitness. Additionally, the structural data and metadata that we record enable the reconstruction and reproducibility of the associated NN.

## Specifications Table

**Table:** 

Subject	Applied Machine Learning
Specific subject area	Neural network metadata and learning curve data
Type of data	Tabular data in TXT files.
How the data were acquired	The neural networks were generated, trained, and validated on the POWER9 Summit supercomputer^1^ using the PyTorch library (v. 1.3.1) and Python language (v. 3.6.10).
Data format	Raw
Description of data collection	The data consist of tables describing NNs and their learning curves. We generate each NN with random parameters and train it on an image dataset for 40 epochs, using stochastic gradient descent and cross entropy loss. For each NN, we record the randomized parameter values and image dataset used for training. Every half epoch throughout raining, we validate the NN and record its fitness.
Data source location	Summit Supercomputer at Oak Ridge National Laboratory Oak Ridge, TN, United States
Data accessibility	Repository name: Harvard Dataverse Data identification number: doi:10.7910/DVN/ZXTCGF Direct URL to data: https://doi.org/10.7910/DVN/ZXTCGF
Related research article	A. Keller Rorabaugh, S. Caíno-Lores, T. Johnston, M. Taufer, Building high-throughput neural architecture search workflows via a decoupled fitness prediction engine. IEEE Transactions on Parallel and Distributed Systems, 2022, In Press. DOI 10.1109/TPDS.2022.3140681[12]

**Value of the Data**

• The ubiquity of NNs has lead to significant investment in tools for NN design [1], [2]. Development of such tools requires knowledge about diverse NNs and their learning curves (i.e., fitness throughout training) [3]. Existing NN repositories store only highly accurate NNs, together with their final fitness values, and do not include the full NN learning curves [4], [5]. Our dataset fills this gap by recording complete learning curves for a wide variety of random NNs.
• Our data is relevant for researchers developing tools for NN design. Such tools include neural architecture search [6], [7], [8] and methods for NN fitness prediction and training termination [9], [10], [11]. Learning curve data is essential to the development of methods for NN fitness modeling and prediction [3], [12].
• Researchers can use our dataset to study evolution of NN fitness during training and identify relationships between an NN’s structure and its fitness on a given image dataset. For example, a researcher can analyze specific columns from each NN table in order to study the relationship between particular design elements of the NNs (e.g. learning rate; batch size; number, order, and type of layers) and the learning curves.
• Parametric modeling of learning curves is increasingly used to model and predict fitness in machine learning applications [3]. Statistical methods can be applied to our dataset to analyze the shape of the learning curves. This enables researchers to identify families of functions that well model such curves and make informed choices about which modeling functions to employ in parametric modeling methods [12].
• Our data can advance effective searches for accurate NNs, which have a far-reaching impact on many fields. Accurate NNs can be used to extract structural information from raw microscopy data [13], detect IO interference in batch jobs [14], predict performance of business processes [15], predict soil moisture or maize yield [16], detect rare transitions in molecular dynamics simulations [17], [18], analyze cancer pathology data [19], and map protein sequences to folds [20].

## 1 Data Description

**Fig. 1:**
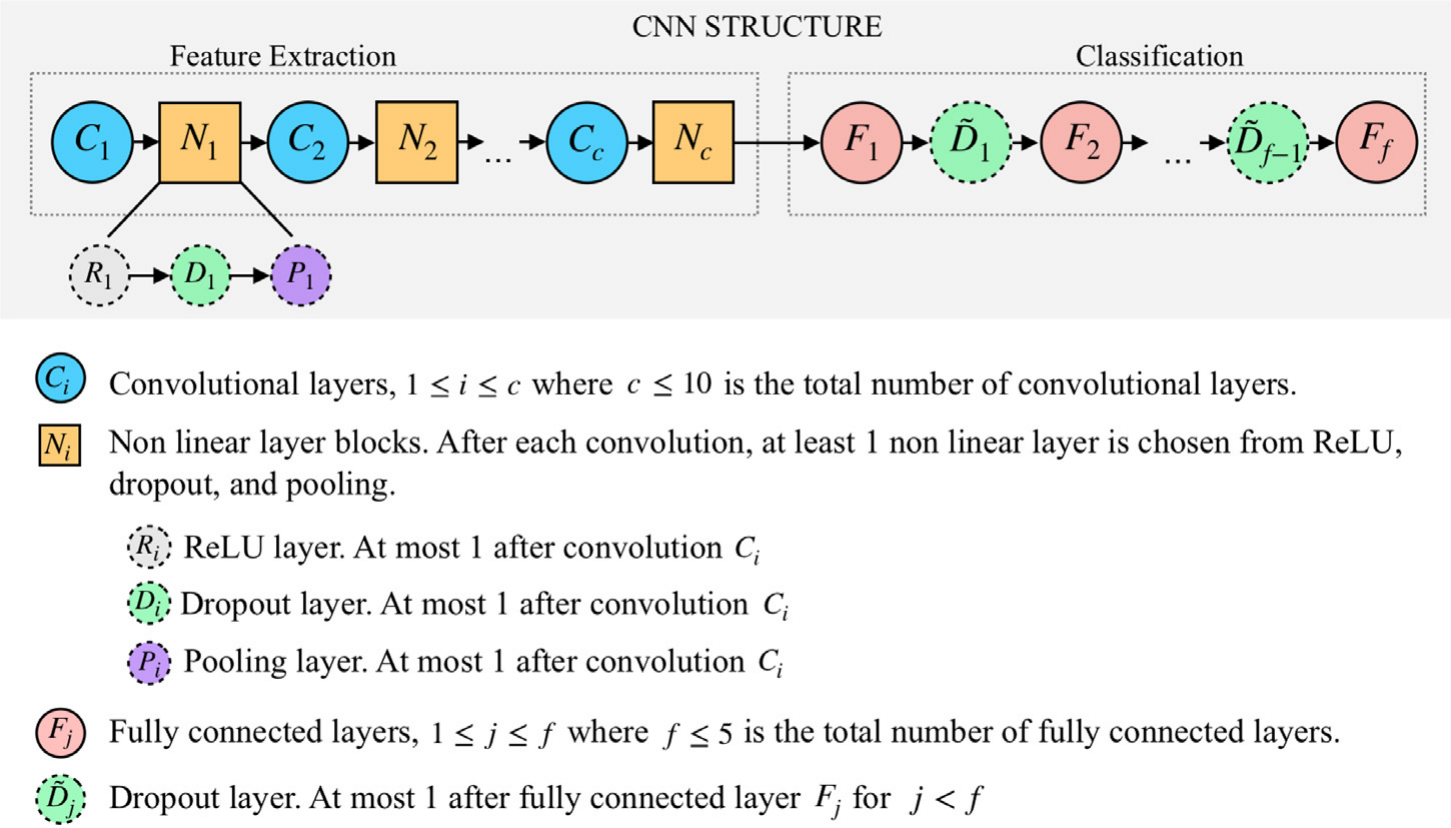
Structure of Generated NNs.

We define a taxonomy of the random NNs that we generated and trained to build our dataset. Fig. 1 depicts the structure of our NNs. Each NN is composed of two sections, *Feature Extraction* and *Classification*. The *Feature Extraction* section of the NN consists of convolutional and non-linear layers; we alternate convolutional layers and non-linear layers such that each convolutional layer is followed by at least one and at most three non-linear layers before any other convolutional layer is applied. The *Classification* section of the NN consists of fully connected layers, with possible dropout layers in between.

Our data depict the lifespan of 6000 NNs throughout generation, training, and validation stages, across 40 epochs of training, with fitness values captured every half epoch. The NNs are randomly generated using our taxonomy. The dataset consists of 6000 tables, each with 28 columns and 81 rows, together with a Python script that demonstrates how to load the data into a Pandas DataFrame and how to calculate and save metrics of interest like mean accuracy or the NN’s learning rate. The dataset is publicly available in the Harvard Dataverse repository: https://doi.org/10.7910/DVN/ZXTCGF. The data format is tabular: the information is organized in .txt files. Each.txt file contains a single table capturing the lifespan of one NN. Each table contains 81 rows and 28 columns. The first row stores the column names, and the remaining 80 rows correspond to every half epoch throughout the lifespan of the NN, beginning at epoch 0.5, and ending at epoch 40. The columns correspond to the fitness data and the metadata that we track throughout the lifespan of the NNs. The first four columns contain training and validation data of the NN; these values change throughout the lifespan of the NN, and hence these columns populate all rows. The remaining columns contain metadata describing the generation of the NN and its structure; these values do not change throughout the lifespan of the NN and thus are only recorded in the second row. From left to right the columns of each NN table are as follows:

1. **epochs**: Elapsed epochs of training.
2. **trainLoss**: Training loss at the given epoch.
3. **valLoss**: Validation loss at the given epoch.
4. **valAcc**: Validation accuracy percentage at the given epoch. Values range between 0 and 100.
5. **ID**: Unique identifier of the NN described in the table.
6. **random_seed**: The random seed used for NN generation.
7. **train_GPU**: A boolean value indicating whether the NN is trained using the GPU.
8. **torch_set_deterministic**: A boolean indicating whether pytorch’s “set_deterministic” flag is activated during training.
9. **dataset**: The name of the image dataset on which the NN is trained.
10. **batch_size**: Batch size used for NN training.
11. **loss_fn**: The loss criterion used to calculate loss during training.
12. **optimizer**: The optimizer used during training.
13. **learning_rate**: The learning rate used for training the NN.
14. **momentum**: The momentum value used for training the NN; if momentum is not used, the value is 0.
15. **dampening**: The dampening value used for training the NN; if dampening is not used, the value is 0.
16. **weight_decay**: The weight decay value used for training the NN; if weight decay is not used, the value is 0.
17. **layer_types**: The type of non-linear layers following each convolution, reported in a hyphen separated list of integers. Each integer corresponds to one convolutional layer, and its value encodes the block of non-linear layers following that convolutional layer. The integers are recorded consecutively, beginning with the integer corresponding to the first convolutional layer. Table 1 depicts the block of non-linear layers encoded by each integer value. For example, layer_types = 1-5-2, would mean the first convolution is followed by a ReLU layer, the second convolution is followed by a ReLU layer and then a dropout layer, and the third convolution is followed by a pooling layer.

**Table 1:** Block of non-linear layers encoded by each integer value.

*integer*	1	2	3	4	5	6	7

*encoded layers*	ReLu	pooling	ReLu, pooling	dropout	ReLU, dropout	dropout, pooling	ReLU, dropout, pooling
18. **dropout_rate**: The dropout rate to use for all dropout layers specified in “layer_types”. If no dropout layers are specified, the value is 0.
19. **convKernels**: The kernels used for each convolutional layer, reported in a hyphen separated list of integers. Each integer is the kernel for one convolutional layer. Kernels are recorded consecutively, beginning with the first convolutional layer.
20. **convStrides**: The strides used for each convolutional layer, reported in a hyphen separated list of integers. Each integer is the stride for one convolutional layer. Strides are recorded consecutively, beginning with the first convolutional layer.
21. **convPaddings**: The padding values used for each convolutional layer, reported in a hyphen separated list of integers. Each integer is the padding value for one convolutional layer. Padding values are recorded consecutively, beginning with the first convolutional layer.
22. **poolKernels**: The kernels used for each pooling layer, reported in a hyphen separated list of integers. Each integer is the kernel for one pooling layer. Kernels are recorded consecutively, beginning with the first pooling layer. If there are no pooling layers in the NN, then this entry is empty.
23. **poolStrides**: The strides used for each pooling layer, reported in a hyphen separated list of integers. Each integer is the stride for one pooling layer. Strides are recorded consecutively, beginning with the first pooling layer. If there are no pooling layers in the NN, then this entry is empty.
24. **poolPaddings**: The padding values used for each pooling layer, reported in a hyphen separated list of integers. Each integer is the padding value for one pooling layer. The padding values are recorded consecutively, beginning with the first pooling layer. If there are no pooling layers in the NN, then this entry is empty.
25. **convFilters**: The number of filters of each convolutional layer, reported in a hyphen separated list of integers. Each integer is the number of filters for one convolutional layer. The number of filters are recorded consecutively, beginning with the first convolutional layer.
26. **FC_dropout_rate**: The dropout rate for dropout layers in the Classification section of the NN; if no dropout layers are added in Classification section, the value is 0.
27. **FC_dropout_layers**: A hyphen separated list of integers denoting which fully connected layers are followed by dropout layers. The integers represent boolean values–0 for False, 1 for True. The integers correspond to consecutive fully connected layers, beginning with the first one, where a value of 0 means the current fully connected layer is not followed by a dropout layer, and a value of 1 means the current fully connected layer is followed by a dropout layer.
28. **FCFilters**: A list of the number of filters of each fully connected layer. The number of filters are reported in a hyphen separated list of integers. Each integer is the number of filters for one fully connected layer. The number of filters are recorded consecutively, beginning with the first fully connected layer.

Our dataset amounts to 109.4MB of data distributed in 6000 tabular files. Because of the significant size of the dataset, we do not include the full dataset in the text of this paper. The full dataset can be downloaded from our public Harvard Dataverse repository; a link is included in the Specifications Table under “Data Accessibility.” Table 2 gives an example of the first 3 rows of one of these 6000 NN tabular.txt files: the first row contains the column names; the second row contains the training and validation data at epoch 0.5 as well as the metadata describing generation of the NN and its structure; the third row contains the training and validation data at epoch 1.0. The remaining 78 rows contain training and validation data for each consecutive half epoch up through 40 epochs; we do not include these rows in the paper because of space constraints. The full table can be found in our dataset. The NN represented in this table has unique ID “2021_02_15_12_18_09_100387” (row 2, column 5 of Table 2) and is trained on CIFAR-100 (row 2, column 9 of Table 2). The path to this table in our dataset is “CIFAR-100_models/2021_02_15_12_18_09_100387.txt”.

**Table 2:** First three rows of an NN table.

	*Column 1*	*Column 2*	*Column 3*	*Column 4*	*Column 5*	*Column 6*	*Column 7*	

*Row 1*	**epochs**	**trainLoss**	**valLoss**	**valAcc**	**ID**	**random_seed**	**train_GPU**	...
*Row 2*	0.5	4.604796782661887	4.582063616775885	1.010404161664666	2021_02_15_12_18_09_100387	3,153,530,971	True	
*Row 3*	1.0	4.604823879167145	4.582446265802151	1.0304121648659463				

		*Column 8*	*Column 9*	*Column 10*	*Column 11*	*Column 12*	*Column 13*	

	...	**torch_set_deterministic**	**dataset**	**batch_size**	**loss_fn**	**optimizer**	**learning_rate**	...
		False	CIFAR100	49	CrossEntropyLoss	SGD	0.01005814462371971	

	*Column 14*	*Column 15*	*Column 16*	*Column 17*	*Column 18*	*Column 19*	

	...	**momentum**	**dampening**	**weight_decay**	**layer_types**	**dropout_rate**	**convKernels**	...
		0	0	0.05815562399917578	1-2-2	0	32-1-1	

		*Column 20*	*Column 21*	*Column 22*	*Column 23*	*Column 24*	*Column 25*	

...	**convStrides**	**convPaddings**	**poolKernels**	**poolStrides**	**poolPaddings**	**convFilters**	...
	9-1-1	0-4-0	9-1	5-1	0-0	304-38-257	

	*Column 26*	*Column 27*	*Column 28*				

...	**FC_dropout_rate**	**FC_dropout_layers**	**FCFilters**				
	0.23811313201015194	0	343-100				

Our dataset includes the python script *DataLoader.py*. This script shows how to load the tabular .txt files into a Pandas DataFrame, isolate columns of interest, perform computations (e.g. calculating max, min, or mean values of the accuracy or loss of an NN over its lifespan), aggregate computations for all NNs into a single DataFrame, and save the aggregate calculated metrics in a .csv file.

## 2 Experimental Design, Materials and Methods

### 2.1 Neural Network Generation

For each of our three image datasets (i.e., CIFAR-100, FashionMNIST, and SVHN) we generate 2000 NNs with random parameter values and train them on that image dataset, for a total of 6000 NNs described in our dataset. We generate each NN according to the structure in Fig. 1 with uniformly randomized parameter values from the intervals defined in Table 3.

**Table 3:** Parameters for NN generation. Values are uniformly randomized in the specified intervals.

Parameter	Values
*Feature Extraction Parameters*	
Number of convolutional layers	[1,10]
Kernel	[1,dimensionofinput]
Stride	[1,kernel]
Padding	[0, 5]
Number of filters	[μ^a^, 400]
Number and type of non-linear layers in blocks $N_{i}$	[1, 30]; ReLU, dropout, pooling
Dropout rate for dropout layers	[0.1, 0.7]
Pool kernel for pooling layers	[1,dimensionofinput]
Stride for pooling layers	[1,poolkernel]
Padding for pooling layers	[0,floor(poolkernel/2)]
*Classification Parameters*	
Number of fully connected layers	[1, 5]
Number of filters	[0, 400]
Dropout rate for dropout layers^b^	[0.1, 0.7]
*Training Parameters*	
Learning rate	$10^{p}$, p∈[−6.0,0.0]
Momentum^b^	$10^{p},p\in \left[-6.0,0.0\right]$
Dampening^b^	$10^{p},p\in \left[-6.0,0.0\right]$
Weight decay^b^	$10^{p},p\in \left[-6.0,0.0\right]$
Batch size	[25, 250]

a Either μ= number of channels or μ= number of filters of $C_{i-1}$, depending on whether increasing number of filters is enforced.

b These parameters are only taken into account if they are randomized to be true.

Zooming into Table 3, we generate three different sets of parameters (i.e., Feature Extraction Parameters, Classification Parameters, and Training Parameters).

**Fig. 2:**
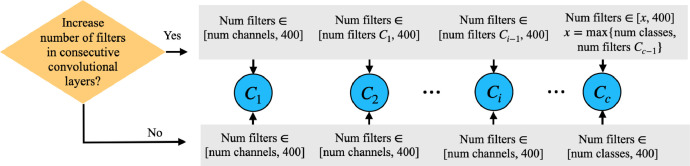
Randomizing number of filters of each convolutional layer.
  As depicted in Fig. 1, each convolutional layer is followed by non-linear layers randomized from the following types: ReLU, dropout, or pooling. We randomize kernel, stride, and padding values for each pooling layer, and we choose a dropout rate to use for all dropout layers in the *Feature Extraction* section.
• Classification Parameters
  We randomize the number of fully connected layers, and we generate a random boolean that determines whether or not to allow any dropout layers between pairs of fully connected layers. If the boolean is True, we randomize the dropout rate to use for all dropout layers in the *Classification* section. Then, for each fully connected layer except the final one, we generate a random boolean to decide whether or not to add a dropout layer after this fully connected layer. If the boolean is False, the dropout rate is 0, and we do not add any dropout layers in the *Classification* section.
• Training Parameters
  We randomize the learning rate, momentum, dampening, and weight decay to use for training the NN. As depicted in Fig. 3a, for the training parameters momentum, dampening, and weight decay, we generate a random boolean to determine whether or not to activate that parameter. If the parameter is not activated, we set the parameter’s value to 0.

• Feature Extraction Parameters
  On each of our three image datasets, we generate 2000 NNs, 200 each with x number of layers, for 1≤x≤10. This ensures that the number of convolutional layers of the NNs is uniformly distributed between 1 and 10. For each convolutional layer, we randomize kernel, stride, and padding values, as well as the number of filters.
  Often, NNs are structured so that the number of filters for the convolutional layers increases with each layer. We generate some NNs whose convolutional filters increase sequentially, but we do not restrict our data to only NNs with this property. We achieve this by generating a random boolean for each NN that determines whether or not to increase the number of filters in each sequential convolutional layer. Fig. 2 shows the process to randomize the number of filters for the convolutional layers of each NN, depending on the value of the boolean and the position of the layer in the NN. If the number of filters is not required to increase, then the number of filters for the last convolution $C_{c}$ is chosen uniformly in the range [*number of classes*, 400], and the number filters for each convolution $C_{i}$, i<c, is always chosen uniformly in the range [*number of image channels*, 400]. Otherwise, if the number of filters is required to increase, then there are three possible cases:
  • The number of filters for the first convolution, $C_{1}$, is always chosen uniformly in the range [*number of image channels*, 400].
  • The number of filters for the last convolution, $C_{c}$, must be at least the number of classes in the image dataset. In addition, the number of filters must also be at least the number of filters of the previous convolution.
  • For the other intermediate layers, the number of filters for $C_{i}$ is chosen uniformly in [*number of filters of* $C_{i-1},400$].

**Fig. 3:**
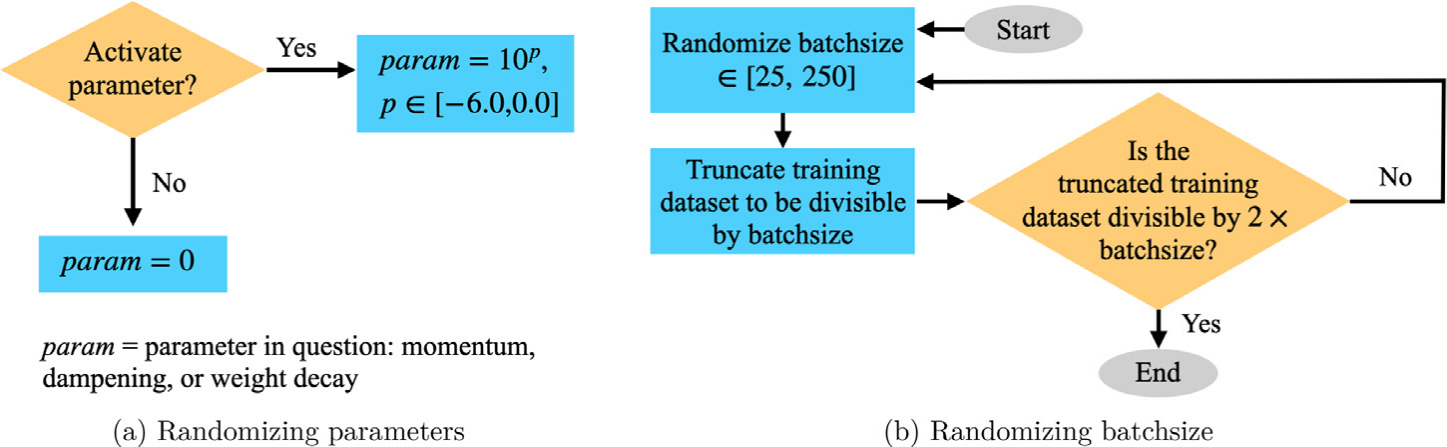
Randomizing training parameters.
  Finally, we randomize the batch size to use for training. The procedure is given in Fig. 3b. We randomize batch size uniformly between 25 and 250 and truncate the training image dataset to be divisible by batch size. Because we validate every half epoch, we also need the number of samples in the truncated dataset to be divisible by twice the batch size. If this divisibility condition is not met, we re-randomize batch size until it is.

### 2.2 Neural Network Training and Validation

As noted earlier, each of our generated NNs is trained on one of three image datasets: CIFAR-100, F-MNIST, and SVHN. Each of these image datasets comes partitioned into training and testing sets; we use the training set for training the NNs and the testing sets for validating the NNs. The CIFAR-100 dataset contains 50,000 images for training and 10,000 images for testing. The F-MNIST dataset contains 60,000 images for training and 10,000 images for testing. The SVHN dataset contains 373,257 images for training and 26,032 images for testing.

Each generated NN trains on the training set of one of these three datasets for a total of 40 epochs. Every half epoch throughout training, the training loss is recorded and training is paused in order to validate the network on the testing set and record the NN’s validation accuracy and validation loss. After validation, training resumes for the next half epoch. All neural networks are trained using stochastic gradient descent. The loss criterion used is cross entropy loss.

## CRediT authorship contribution statement

**Ariel Keller Rorabaugh:** Methodology, Software, Validation, Investigation, Data curation, Writing – original draft, Writing – review & editing. **Silvina Caíno-Lores:** Conceptualization, Methodology, Writing – original draft, Writing – review & editing. **Travis Johnston:** Conceptualization, Methodology. **Michela Taufer:** Conceptualization, Methodology, Supervision, Writing – review & editing, Funding acquisition.

## Declaration of Competing Interest

The authors declare that they have no known competing financial interests or personal relationships that could have appeared to influence the work reported in this paper.

## Footnotes

1 https://docs.olcf.ornl.gov/systems/summit_user_guide.html#system-overview
